# Improved 3D image reconstruction via deep-learning-based fusion of light-field microscopy and Fourier light-field microscopy images

**DOI:** 10.1117/1.JBO.31.3.036002

**Published:** 2026-03-03

**Authors:** Er Ouyang, Liang Liang, Xuhui Zhou, Lin Zhao, Hui Fang

**Affiliations:** a Shenzhen University, Institute of Microscale Optoelectronics and Shenzhen Key Laboratory of Microscale Optical Information Technology, Nanophotonics Research Center, Shenzhen, China; b Hunan Institute of Science and Technology, Yueyang, China

**Keywords:** light-field microscopy, Fourier light-field microscopy, deep learning, image fusion, 3D reconstruction, hierarchical cascade

## Abstract

**Significance:**

Light-field microscopy (LFM) is a scanning-free 3D imaging technique that is useful for observing dynamic biological systems due to its unique capability to capture both spatial and angular information from samples in a single exposure. However, LFM suffers from the spatial–angular information trade-off associated with microlens arrays, and its spatial resolution is usually unsatisfactory for fine-structure imaging.

**Aim:**

To overcome this bottleneck, we introduce a deep-learning-based image fusion technique that combines LFM images with Fourier LFM (FLFM) images. The high spatial resolution of FLFM is combined with the dense angular acquisition capability of LFM to improve 3D image reconstruction quality.

**Approach:**

The deep learning network was trained with LFM, FLFM, and epipolar plane image data. The proposed neural network employs specialized feature extraction modules for each modality, with a U-Net backbone for 3D reconstruction, and integrates a hierarchical cascade-based result-level fusion strategy to jointly optimize multimodal features. This approach significantly enhances detail preservation and depth recovery in the final output.

**Results:**

Results obtained using a publicly available dataset of synthetic tubulins demonstrate that the proposed method outperforms state-of-the-art techniques. Quantitatively, it achieved a peak signal-to-noise ratio (PSNR) of 38.4729 and a structural similarity index measure (SSIM) of 0.9876, significantly outperforming both traditional algorithms and single-modality deep learning approaches. Furthermore, validation on a mouse brain blood vessels dataset confirms the effectiveness of the method in reconstructing biological structures, achieving a PSNR of 35.0548 and an SSIM of 0.8424.

**Conclusions:**

We introduce an approach that combines LFM with FLFM, providing an efficient and reliable solution for practical LFM applications. The deep-learning-based framework demonstrates significant potential to simultaneously accelerate imaging acquisition and enhance 3D reconstruction quality, offering further possibilities for computational microscopy.

## 1 Introduction

Light-field microscopy (LFM),^1^ which involves placing a microlens array (MLA) on the intermediate image plane, transforms a conventional bright-field microscope into a scanning-free system and is an established high-speed 3D imaging technique. By capturing the spatial and angular information of a sample in a single exposure, LFM enables efficient 3D reconstruction of the targeted volume.^2^ LFM offers important advantages over traditional scanning-based 3D imaging methods, including faster acquisition, lower storage requirements, and minimal phototoxicity; hence, it is particularly suitable for long-term live biological imaging.^3^^,^^4^ Because of these benefits, LFM is widely used to observe dynamic biological systems, such as neural activity and developmental processes. However, this technique still has certain limitations in practical applications, such as insufficient reconstruction accuracy, which leads to detail loss and artifacts;^5^ inadequate anisotropic spatial and depth resolutions;^6^ and computationally intensive iterative reconstruction processes.^7^

To overcome the spatial resolution problem, Fourier light-field microscopy (FLFM) has recently been developed,^8^ which repositions the MLA from the image plane to the back focal plane. By utilizing larger-aperture MLAs, FLFM achieves spatially invariant sampling and improved spatial resolutions while retaining the single-shot advantages of conventional LFM.^9^ However, it sacrifices depth information because of the considerably reduced angular sampling. Moreover, similar to LFM, FLFM also suffers from insufficient anisotropic spatial and depth resolutions and reconstruction artifacts, which limit precision in biomedical and materials science applications.^10^ These persistent challenges stem from attempting to use either modality alone to solve problems that require both angular and spatial precision. Herein, we propose an image fusion approach that combines the two modalities to improve 3D image reconstruction quality.

Conventional LFM-based 3D image reconstruction techniques mainly employ iterative model-based methods that utilize a point-spread function (PSF) to represent the optical imaging process, with deconvolution techniques^11^^,^^12^ being used to improve spatial resolution. Although these methods have been progressively refined,^13^^–^^15^ they remain fundamentally constrained by their inherent computational complexity and performance limitations. However, the advent of deep learning has revolutionized LFM reconstruction paradigms, providing new opportunities to address the unresolved challenges. For instance, CNN-based Richardson-Lucy (RL) deconvolution has been employed to optimize LFM image reconstruction.^16^ In 2021, Vizcaino et al.^17^ proposed the LFM-Net algorithm, which leverages the U-Net^18^ architecture to combine LFM with experimental datasets, improving reconstruction performance and speed. Wang et al.^19^ developed a deep neural network called VCD-Net, which significantly enhances the resolution of LFM by exploiting frequency aliasing between different angles in LF data. Although these deep learning methods have advanced LFM imaging, their inherent limitations in capturing comprehensive detail and depth information remain unaddressed.

The LF image fusion technique represents an innovative approach to solving the aforementioned resolution and reconstruction accuracy problems. Thus far, extensive research has been conducted on image fusion from LF cameras,^20^^–^^23^ typically using a dual-modal approach that combines an LF camera with a conventional camera to extract extra information with a much higher spatial resolution. The same approach has been utilized in LFM-based image fusion, although this research field is still in its nascent stage. Geng et al.^24^ designed a hybrid LF imaging system to reconstruct high-resolution LF images from synchronously recorded high-resolution 2D images and low-resolution 4D LF data, achieving high-speed visualization of biological dynamics. Lu et al.^25^ combined high-resolution images from a standard microscope with LFM images to reconstruct a high-resolution map via phase-space retrieval. Liu et al.^26^ fused wide-field images with FLFM images, significantly improving the resolution of FLFM. The aforementioned research has verified the potential of fusion technology to optimize the imaging quality of LF microscopy. However, Eckstein et al.^27^ highlighted that the improvement in LF quality is not entirely due to the fusion with conventional images; rather, it stems from the effect of an intermediate interpolation step in the LF itself. Although this interpolation can partially compensate for the drawback of the sparse sampling of the LF, it is essentially a linear or low-order nonlinear approximation; hence, it cannot fully capture the complex correlations within the high-dimensional LF data.^28^^,^^29^

Notably, the information captured in LFM images and FLFM images is complementary in nature; although LFM offers high angular resolution, which is effective in capturing optical information from multiple perspectives, its spatial resolution is relatively low; conversely, FLFM offers higher spatial resolution, enabling more detailed reconstruction of sample structures,^30^^,^^31^ albeit with much less angular sampling. These complementary properties can be manifest distinctly in their respective epipolar plane images (EPIs), which describe the trajectory of an object in different views.^32^ Specifically, EPIs generated via LFM exhibit accurate depth-gradient representation due to the denser angular sampling, whereas those derived via FLFM have sharper structural edges that enable more robust feature extraction. Many LF-related studies have utilized EPIs to improve image reconstruction quality. Zhang et al.^33^ proposed leveraging the linear structures in EPIs and local linear embedding for LF depth estimation. Liu et al.^34^ introduced multi-angle epipolar geometry constraints into LF reconstruction, leveraging angular geometric features to recover fine texture details. Song et al.^35^ incorporated EPI-based physical priors into a regularization framework, modeling depth-dependent shift invariance in phase space via linear convolution and exploiting spatial sparsity and inter-class relationships with soft labels. Van Duong et al.^36^ developed an efficient CNN architecture that jointly exploits spatial–angular correlations and multi-orientation EPI features using specialized extractors and adaptive fusion, demonstrating robust performance across both narrow-baseline and wide-baseline LF datasets. Rossi et al.^37^ proposed an LF super-resolution network based on spatial–angular and multimodal EPI representation fusion, which decomposes the LF into EPI stacks along four view directions. Their approach employs a directional EPI feature extractor to model the linear structures of EPIs, which allows for capturing subpixel-level depth-gradient information. Building upon these EPI-based approaches and recognizing the complementary properties of LFM and FLFM EPIs, we developed a neural network architecture that strategically fuses EPI representations from both modalities.

Inspired by the success of VCD-Net in LFM and FVCD-Net^38^ in FLFM, as well as by the progress of LFM-based image fusion technology, we propose a multimodal LFM 3D reconstruction algorithm to overcome the limitations of single-modal methods. The key component of this algorithm is a two-stage hierarchical result-level fusion strategy for multimodal fusion. First, the LFM and FLFM data are processed separately through reconstruction networks to generate an initial 3D reconstruction. Simultaneously, EPIs are extracted via LFM and FLFM for disparity analysis and depth feature extraction, respectively. Subsequently, the LFM reconstruction results are fused with the corresponding EPI features through a fusion block, whereas the FLFM reconstruction results are combined with their respective EPI features via a similar fusion block. Finally, cross-modal optimization is performed to integrate the fusion results from both modalities. By fusing the reconstruction results of each modality step by step, a high-resolution 3D reconstruction is realized. We used the synthetic micro-protein tube data set provided by VCD-Net^19^ as the benchmark data for our experiments. The main contributions of this study are as follows:

1. A multimodal fusion-based reconstruction algorithm that combines LFM images with FLFM images is proposed. By combining the high spatial resolution of FLFM with the disparity information of EPIs from LFM and FLFM, angular–spatial–depth cross-dimensional fusion is achieved, which significantly improves overall reconstruction accuracy.
2. A feature extraction and fusion strategy is designed for LFM images, FLFM images, and EPIs. A result-level fusion strategy is utilized to achieve efficient alignment and complementarity between modal features, which substantially enhances detail restoration in complex scenes.
3. Experiments on the synthetic tubulin dataset and the mouse brain blood vessels dataset demonstrate that the proposed method outperforms existing single-modal methods. Notably, it excels in terms of detail reconstruction, depth consistency, and artifact suppression, providing reliable technical support for 3D imaging applications involving LFM.

## 2 Methods

### 2.1 Overall Strategy

**Fig. 1:**
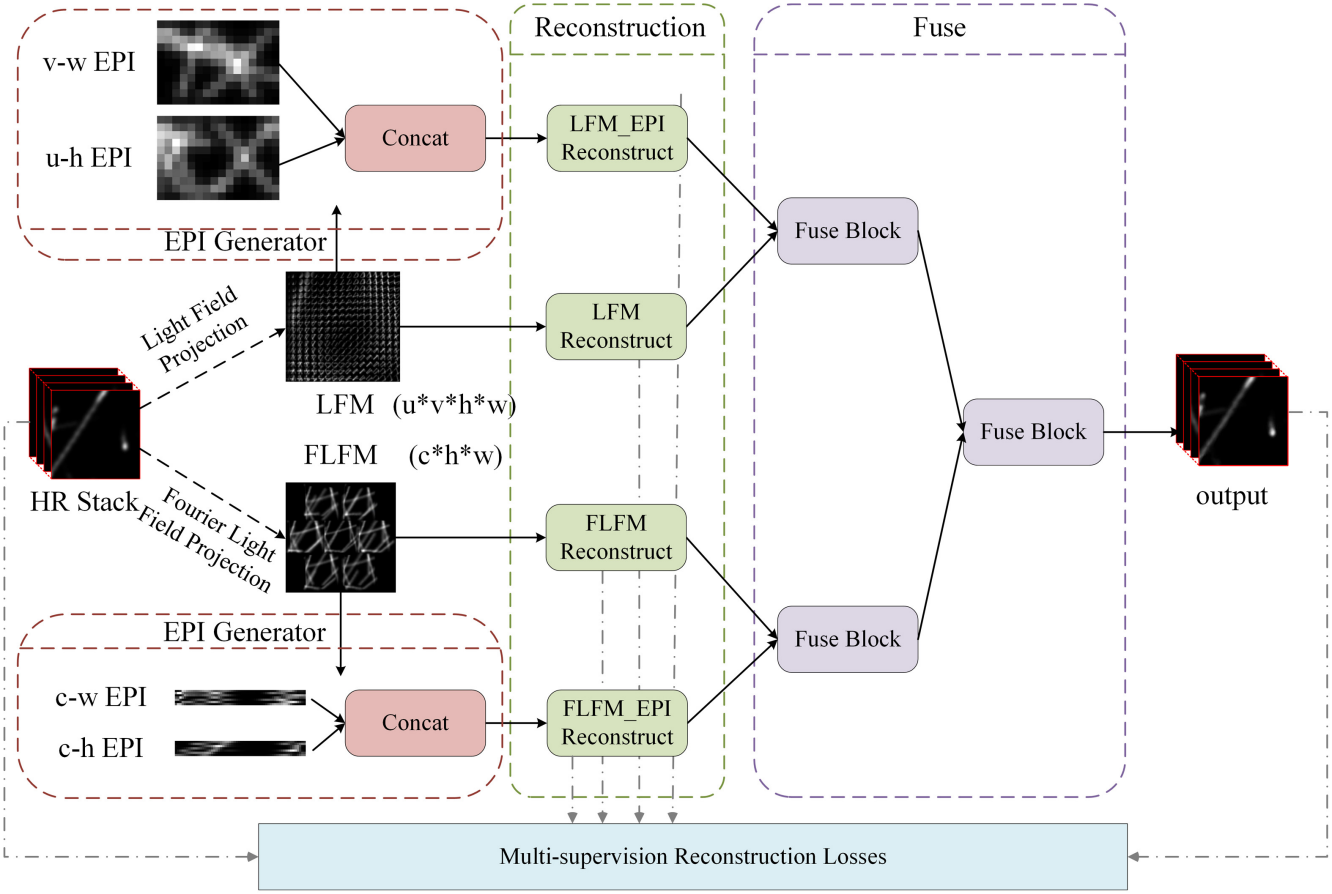
Flowchart of the overall 3D reconstruction process.

To address the challenges of balancing the spatial and angular resolutions and accurately estimating depth information in LFM reconstruction, the proposed algorithm fuses features from LFM images, FLFM images, and EPIs; the reconstruction process is illustrated in Fig. 1. First, high-resolution stack images, obtained from a synthetic tubulin confocal imaging dataset,^19^ are used to generate LFM and FLFM images. From the LFM images, EPIs are extracted to analyze the disparity slopes and obtain a straightforward representation of the sample depth information, which serves as the multimodal input data. Similarly, for the FLFM images, we generate EPIs to enhance the depth estimation accuracy further. Next, a four-branch network is constructed to process the LFM images, FLFM images, and EPIs separately for 3D image reconstruction. Finally, a fusion module integrates the results from the four branches, maximizing the informational advantages of the images from each modality to produce a comprehensive final 3D reconstruction. Regarding the loss function design, the multisupervised reconstruction loss is employed to constrain the outputs of the different branches, which optimizes the reconstruction performance. The overall 3D reconstruction process can be divided into three parts: the simulation-based generation of multimodal input data, the construction of the four-branch reconstruction network, and fusion-based multimodal reconstruction.

### 2.2 Multimodal Simulation Image Generation

**Fig. 2:**
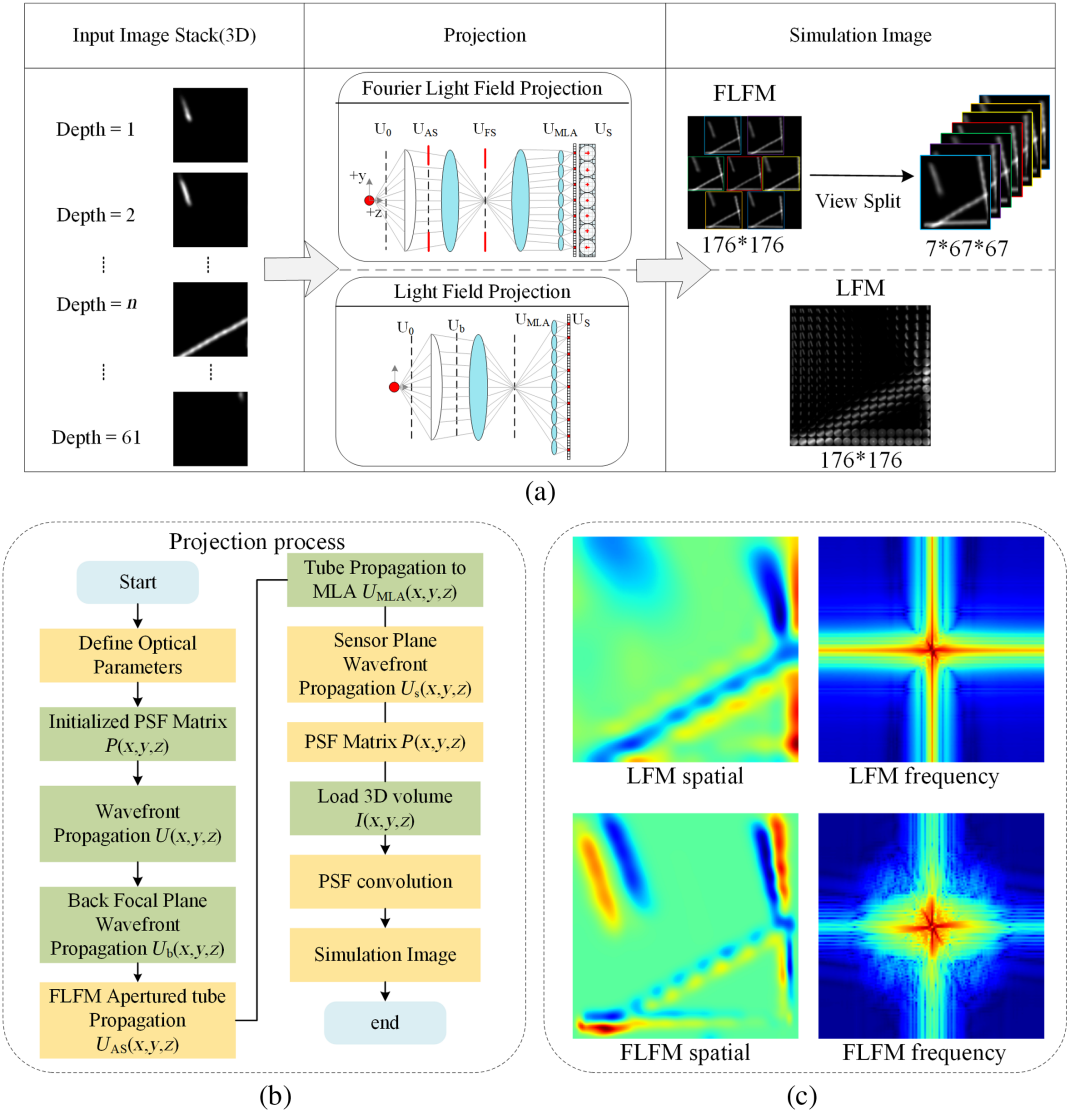
Flowchart of multimodal image generation. (a) Schematic of the optical path principles for simulating LFM and FLFM images. (b) Algorithmic flow of the LFM and FLFM projection processes. (c) Spatial and frequency representations of LFM and FLFM.

To simulate LFM and FLFM images, we utilized an existing process,^19^^,^^38^ which is illustrated in Fig. 2(a). The key differences between LFM and FLFM lie in their optical configurations and wavefront modulation strategies. In LFM, the MLA is placed directly on the native image plane of the objective lens to sample the complete wavefront, which results in a compact system but with inherent angular–spatial coupling. By contrast, FLFM employs a 4f optical system (where f denotes the focal length), where the MLA is positioned on the Fourier plane (conjugate to the back focal plane of the objective lens), which enables aperture clipping in the Fourier domain. This 4f configuration allows FLFM to selectively filter high-frequency components through spatial modulation on the Fourier plane and thereby decouple angular and spatial information while improving axial resolution. These fundamental differences necessitate distinct PSF computation methods. As shown in Fig. 2(b), the algorithmic implementation differences are as follows:

1. Input parameters: LFM requires the numerical aperture (NA), the magnification (M) of the optical, the focal length (f), the depth range (z), the sensor pixel size, and other basic parameters of the system, whereas FLFM also needs aperture function parameters, clipping radius. It should be noted that to ensure each modality could be optimized under ideal conditions, the parameters for LFM and FLFM were adopted from separate systems derived from Refs. 19 and 38. Specifically, for the LFM simulation: the objective has NA = 0.8, M = 40×, refractive index 1.33; the microlens pitch is 150  μm, and the microlens focal length ($f_{\mathrm{ml}}$) is 3500  μm; the image depth of field (DOF) is much larger than the sample thickness. For FLFM: the objective has NA = 1.4 and M = 100×; the tube lens and Fourier lens focal lengths are $f_{R1}=180\;\mathrm{mm}$ and $f_{R2}=300\;\mathrm{mm}$, respectively; the Fourier MLA has a pitch d=3.25  mm, an f-number of 37, and a focal length $f_{L}=120\;\mathrm{mm}$; the DOF for this FLFM system was ∼7  μm.
2. Wavefront propagation model: The wavefront at the back focal plane of the objective lens is calculated from the light intensity $U_{0}(x,y;O)$ at the object plane using a Debye scalar integral representation:^39^
  $$U_{b}(r_{b};O)=\int_{0}^{\theta }U_{o}(\theta ;O)J_{0}(\frac{2\pi }{\lambda }r_{b}\;\sin\;\theta )\sin\;\theta d\theta ,$$
  where $r_{b}$ represents any point $\left(x_{b},y_{b}\right)$ on the plane; λ is the wavelength of the assumed monochromatic light (λ=580  nm in the simulation for LFM; λ=510  nm for FLFM); θ is the aperture angle, which is related to the NA of the objective lens; $J_{0}$ is the zeroth-order Bessel function. In LFM, the full Debye wavefront propagates directly to the MLA plane through optical relay magnification, followed by MLA transmission function T(x,y) calculation, and the wavefront modulation $U_{\mathrm{MLA}}$. However, the FLFM wavefront passes through the aperture limitation of the back focal plane, which introduces aperture clipping in this plane. The wavefront distribution is defined as
  $$U_{AS}(x,y;O)=U_{b}(x,y;O)\cdot P(x,y),$$
  where $P(x,y)=e^{-(\frac{\rho }{\lambda_{c}}{)}^{2\alpha }}$ is the aperture transmission function, which is used to tailor the spatial distribution of the wavefront, and ρ represents the normalized radial coordinates.
3. Diffraction calculation: In LFM, Rayleigh–Sommerfeld diffraction^40^ is directly applied to allow the LF to propagate into the sensor plane $U_{S}$:
  $$U_{s}(x,y;O)=F^{-1}\{F\{U_{\mathrm{MLA}}(x,y;O)\}\cdot e^{i\frac{2\pi }{\lambda }f_{ml}\sqrt{1-(\lambda f_{x}{)}^{2}-(\lambda f_{y}{)}^{2}}}\},$$
  where F and $F^{-1}$ represent the Fourier transform and inverse Fourier transform, respectively; $\left(f_{x},f_{y}\right)$ is the spatial frequency of the imaging plane; and $f_{ml}$ is the focal length of the MLA ($f_{ml}=3500\;\mu m$ for LFM and 120 mm for FLFM in the simulation). By comparison, FLFM requires additional frequency-domain processing, including Fourier transform-based band-limiting:
  $$U_{s\_FLFM}(x,y;O)=F^{-1}\{F[U_{AS}(x,y;O)]\}⊙F[\sin\;c(2\lambda_{c}\rho )]\},$$
  where ⊙ denotes element-wise multiplication, whereas the *sinc* filter explicitly controls frequency-domain aliasing by suppressing spatial frequencies.
4. Optimization strategy: LFM exploits optical symmetry (PSF(x,y,z)=PSF(−x,−y,z)) to compute only half of the PSFs, whereas FLFM requires iterative optimization of P(x,y) through gradient descent:
  $$P^{n+1}=P^{n}+\eta \nabla J(P^{n})\;J(P)=\|{\mathrm{PSF}}_{\text{target}}-{\mathrm{PSF}}_{\text{sim}}{\|}_{2}^{2}+\lambda_{1}\|\nabla P{\|}_{1},$$
  where ${\mathrm{PSF}}_{\text{target}}$ is the ideal diffraction-limited PSF calculated based on scalar theory, ${\mathrm{PSF}}_{\text{sim}}$ is the PSF under the current P(ρ), and $\lambda_{1}$ is a regularization parameter used to enforce smoothness.
5. PSF matrix dimensions: In LFM, the PSF explicitly models the complete angular–spatial response of each point source on both the MLA and sensor planes, generating a 5D tensor with dimensions (61, 16, 16, 11, 11) in the LFM setup. In FLFM, the PSF disregards explicit angular–spatial storage and instead reconstructs the 3D spectrum by utilizing weighting coefficients to combine the Fourier slices. The angular information is converted into phase gradients in the frequency domain through the 4f system’s Fourier filter, which enables the complete wavefront data compression into depth-dependent coefficients with dimensions (61,1,1).
6. Convolution: LFM involves slicing and decomposing a 3D volume by depth and performing spatial convolution between each slice and the PSF for the corresponding depth:
  $$I_{\mathrm{LFM}}(x,y)=\underset{z}{\sum }I(x,y;O)*{\mathrm{PSF}}_{\mathrm{LFM}}(z,w,h,u,v).$$

The convolution in FLFM is a frequency-domain integration rather than a traditional spatial operation. It involves decomposing 3D objects into depth slices, performing a 2D Fourier transform on each slice, and then multiplying the slices by the PSF coefficients, as follows:

$$I_{\mathrm{FLFM}}=F^{-1}\{\underset{z}{\sum }w(z)F\{I_{z}\}(f_{x},f_{y})*{\mathrm{PSF}}_{\mathrm{FLFM}}(f_{x},f_{y},z)\},$$

where w(z) represents the weight coefficient, which is a complex number.

7. Simulated image: After the convolution is completed, LFM directly acquires the 4D simulated image; the perspective map can be obtained based on the geometric arrangement of the MLA. By contrast, FLFM produces 3D Fourier-domain slices $\left(\theta ,f_{x},f_{y}\right)$ via Fourier-plane modulation, with each viewing angle corresponding to distinct spatial-frequency bands determined by the aperture-clipping function. For both modalities, the multidimensional data are subsequently projected onto a 2D sensor plane to generate the final images, which serve as the multimodal input data.

To highlight the characteristic differences between LFM and FLFM imaging modalities, we selected an image pair and performed principal component analysis (PCA) dimensionality reduction^41^ to enable comparative spatial-spectral analysis. As shown in Fig. 2(c), the spatial representation of the LFM image exhibits smooth continuity but relatively low spatial resolution, which renders it suitable for capturing the global structure of the sample. By contrast, the FLFM image provides more detailed information due to its higher spatial resolution, preserving finer structural details that LFM cannot capture. These observations are corroborated by the corresponding frequency spectral maps: LFM predominantly retains low-frequency content, which reflects the global structure, whereas FLFM captures a broader spectrum of frequencies, with a strong emphasis on high-frequency components that enhance the resolution and detail retention. This comparison underscores the complementary strengths of LFM and FLFM, with the former excelling in continuity and the latter in detail accuracy.

**Fig. 3:**
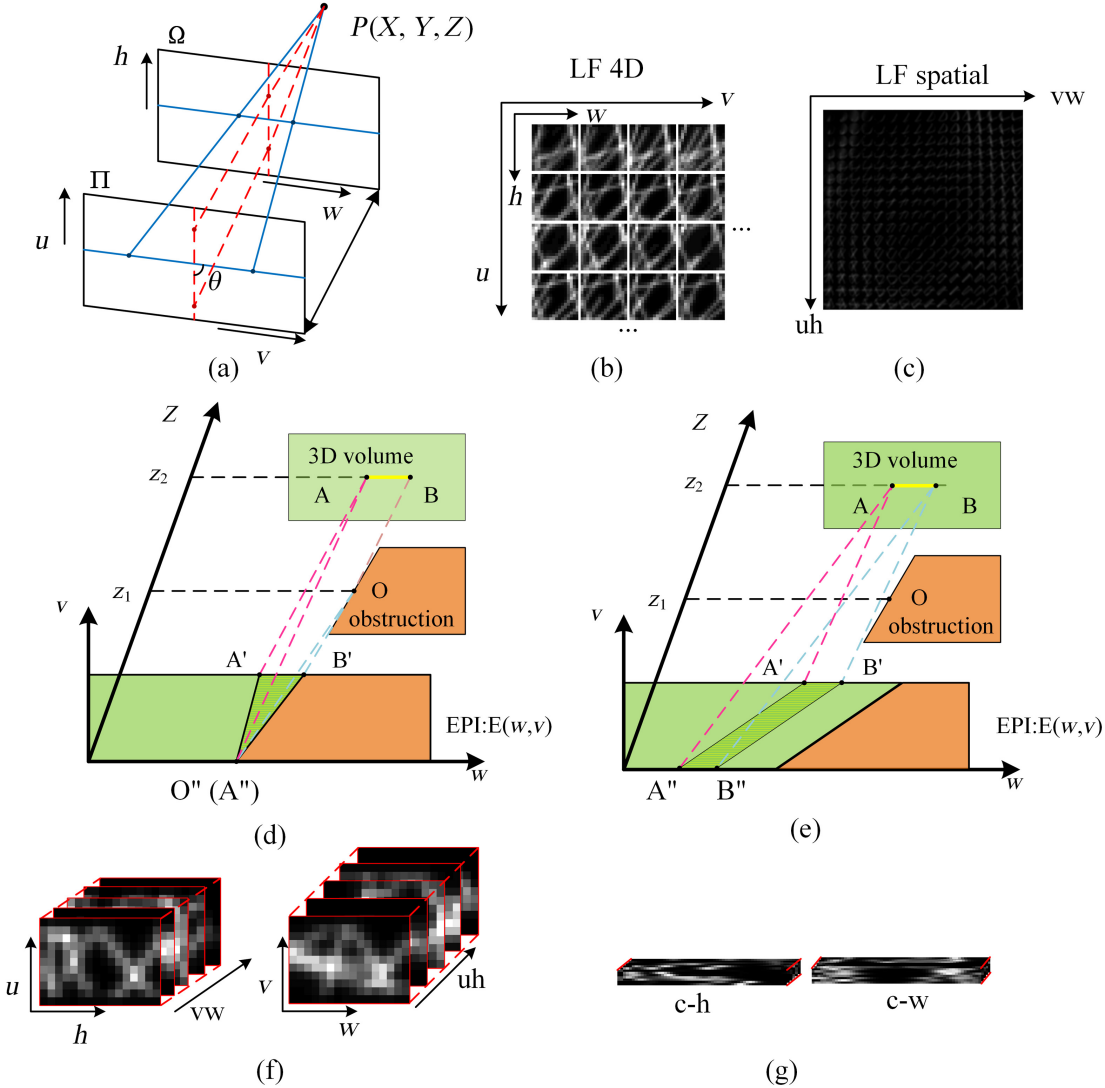
Epipolar plane image generator. (a) Two parallel planes in LFM; (b) 4D representation of LFM; (c) 2D representation of LFM; (d) EPI occlusion relationship for θ=0  deg; (e) EPI occlusion relationship for θ=90  deg; (f) LFM EPI pattern; (g) FLFM EPI pattern.

To parameterize rays from a 3D scene in LFs, two parallel planes are typically used, namely, a spatial plane (Ω) and an angular plane (Π), as shown in Fig. 3(a). The rays are mapped onto two planes, with each ray described by four parameters. In LFM, the captured image is a 4D tensor L(u,v,w,h), where (u,v) and (h,w) represent the angular and spatial coordinates, respectively.^42^ Each angular position (u,v) corresponds to a sub-aperture image [Fig. 3(b)], and merging these images yields a macroscopic angular map [Fig. 3(c)]. The EPI emerges as a crucial analytical tool because of its ability to reveal occlusion relationships in 3D space by fixing one spatial dimension and recording angular variations.^43^ To illustrate occlusion relationships, we consider a 3D volume with an object at $z_{2}$ and an occluder at $z_{1}$ [Fig. 3(d)]. At θ=0  deg, the EPI projection of line segment AB forms the observed shadow triangle, where the slope of AA′ encodes disparity information [Fig. 3(d)]. Adjusting u at θ=90  deg reveals a parallelogram [Fig. 3(e)], which indicates unobstructed visibility. These observations highlight the effectiveness of EPIs in occlusion analysis and depth estimation. The differences between the EPI representations of LFM and FLFM illustrate their complementary strengths, as shown in Figs. 3(f) and 3(g). For LFM, the EPI shows higher angular resolution (dense u-sampling), enabling continuous depth variation for stable depth estimation; however, its lower spatial resolution (sparse w-sampling) limits spatial resolution. By contrast, the EPI of FLFM achieves clearer spatial details due to its higher spatial resolution, although its lower angular resolution results in discrete depth variations. Therefore, integrating the spatial information of FLFM into the LFM enables superior 3D reconstruction performance by combining their complementary strengths.

### 2.3 Multimodal LF Reconstruction Network Based on U-Net

**Fig. 4:**
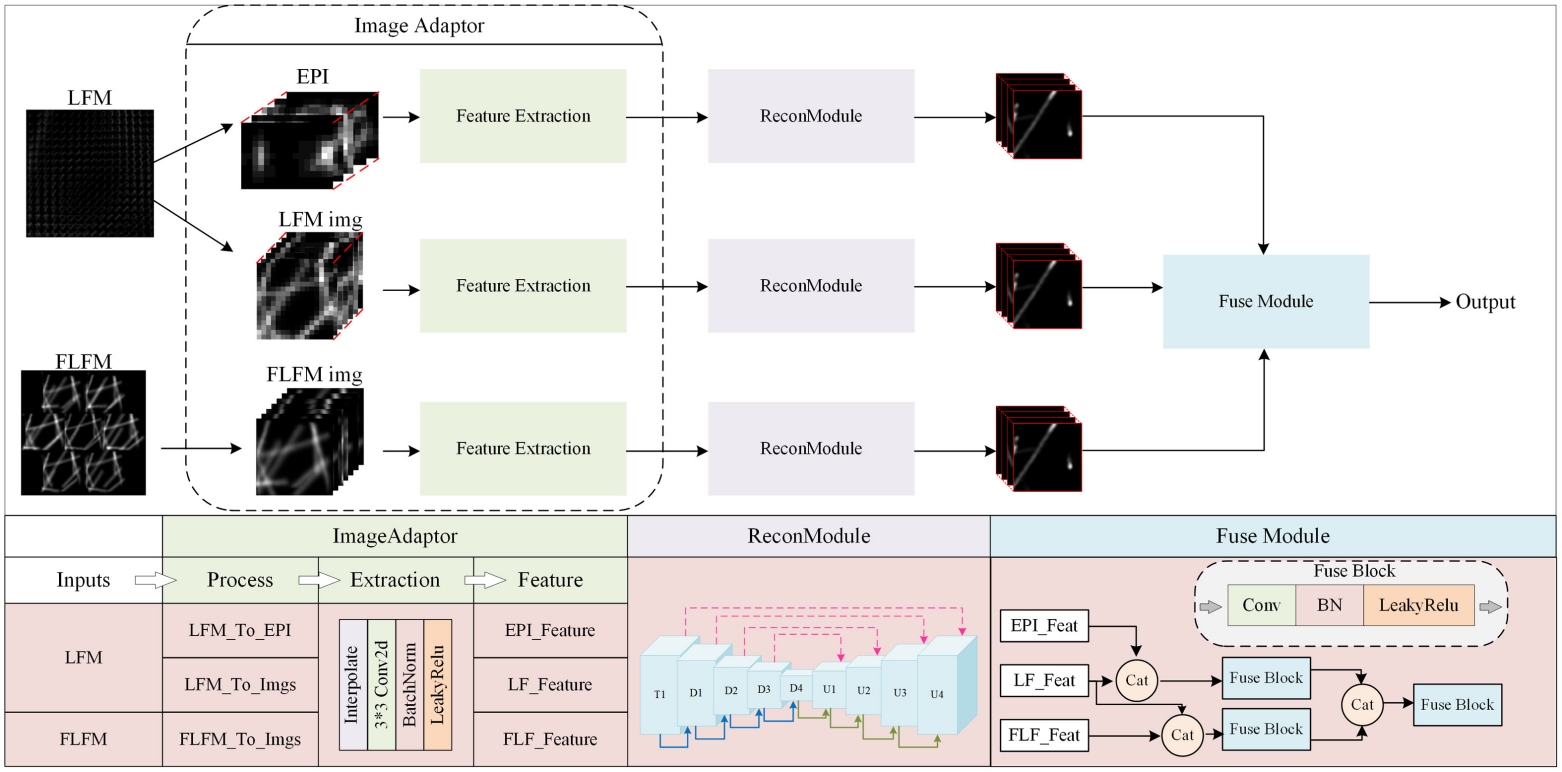
Multimodal reconstruction network.

The proposed 3D reconstruction network utilizes the U-Net architecture to reconstruct LF data. The network comprises three main parts: the adapter module, the reconstruction module, and the fusion module; its structure is shown in Fig. 4. The simulated LFM and FLFM images constitute the input to the network. Due to the differences in dimensionality and features between the modalities, distinct processing methods are required for each modality. The 2D image (u*v,w,h) extracted from the 4D LF data (u,v,w,h) serves as the input to the LFM branch for capturing the angular and spatial information of the light rays. The LFM EPI (LFM_EPI) is formed by converting the 4D data into the 2D u-h plane (v*w,u,h) and v-w plane (u*h,v,w), which is then used as the input to the LFM EPI branch to capture parallax information. Depending on the transmission position of the simulated MLA, the 2D FLFM is cropped into multiple sub-aperture images corresponding to distinct viewing angles (where the number of angles is 2-3-2), which are input to the FLFM branch to increase the spatial resolution. The FLFM EPI (FLFM_EPI) is formed by converting the 3D data into the 2D c-w plane (h,c,w) and c-h plane (w,c,h), which is then used as input to the FLFM EPI branch to capture the parallax information. The images from these modalities are input into the four-branch adapter, which mainly performs interpolation and convolution. Through interpolation, the dimensional spaces of the images from each modality are scaled to a uniform size, and data with different resolutions are aligned to the target resolution; thus, the network maintains a consistent dimension during the fusion step. Then, convolution is performed to extract the features in each modality and unify the channel layers to a fixed dimension.

In the reconstruction module, a U-Net architecture with four branches is utilized to reconstruct the target volume. Within an encoder–decoder framework, the encoder extracts features via multiple convolutional layers, whereas the decoder gradually restores these features to their original spatial structure.

The primary task of the fusion module is to integrate features from different modalities to enhance the quality of the reconstructed images. Inspired by the late-fusion strategies used in multimodal learning,^44^^,^^45^ we propose a hierarchical cascade–based result-level fusion approach. First, the LFM and LFM_EPI branches are fused, along with the FLFM and FLFM_EPI branches. The LFM_EPI and FLFM_EPI branches provide parallax information to the LF branch and the FLFM branch, respectively, to facilitate the reconstruction of depth information. Finally, the two results are combined to improve the reconstruction quality through angle–depth–space cross-modal fusion.

### 2.4 Loss Function

To optimize the reconstruction performance, we introduce a multilevel supervision loss function.^46^ The network not only focuses on the final fused output but also applies supervision loss to the intermediate outputs of each branch. This multilevel loss mechanism ensures that each branch learns features consistent with the ground truth (GT) structure, thus progressively enhancing reconstruction quality. Specifically, the reconstruction results of each branch are compared with the GT 3D structure to compute the respective supervision losses. The mean squared error is used as the loss function. In addition, a global fusion loss is computed between the final 3D reconstruction generated by the fusion module and the GT 3D structure. Therefore, the total loss function is the sum of the individual loss functions, as expressed below:

$$\text{Loss}={\text{loss}}_{\mathrm{LFM}}+{\text{loss}}_{\mathrm{FLFM}}+{\text{loss}}_{\mathrm{LFM}\_\mathrm{EPI}}+{\text{loss}}_{\mathrm{FLFM}\_\mathrm{EPI}}+{\text{loss}}_{\text{Fuse}}.$$

This multilevel supervision loss mechanism progressively reduces the errors in each modality and refines the results obtained after fusion, thereby improving the overall reconstruction accuracy significantly.

## 3 Experiments and Results

### 3.1 Experimental Setup and Dataset

We conducted our experiments using the PyTorch framework, with all training performed on an NVIDIA RTX 4090 GPU to ensure fast and efficient computation. We used a publicly available dataset^19^ containing 3D images of synthetic tubulins, each with a size of 176×176×61  pixels. This dataset was generated from confocal microscopy data of a sparsely labeled tubulin sample imaged with a 40× objective, with XY and Z resolutions of 0.34 and 1  μm, respectively. With an axial sampling interval of 1  μm and a depth range of 61 layers (from −30 to 30  μm), resulting in an imaged sample volume of 59.84×59.84×61  μm. From this confocal dataset, we synthesized both light field and Fourier light field images for our experiments. To comprehensively evaluate the performance of the proposed reconstruction method, the dataset was divided into training and testing subsets in an 8:2 ratio. The test dataset included 472 images, which were used to validate the reconstruction performance of the model. In addition, a mouse brain blood vessels dataset^17^ was also employed to validate the reconstruction capabilities on complex biological tissues.

### 3.2 Evaluation Metrics

To quantitatively evaluate the reconstruction performance of the proposed network, we adopted the peak signal-to-noise ratio (PSNR) and the structural similarity index measure (SSIM) as the evaluation metrics. PSNR measures the similarity between the reconstructed result and the GT, whereas SSIM evaluates the structural fidelity of the reconstructed image, focusing on brightness, contrast, and structural information. These metrics collectively assess the performance of networks in terms of reconstruction quality, structural consistency, and detail preservation, providing a comprehensive evaluation of overall effectiveness.

### 3.3 Comparative Experiments

**Fig. 5:**
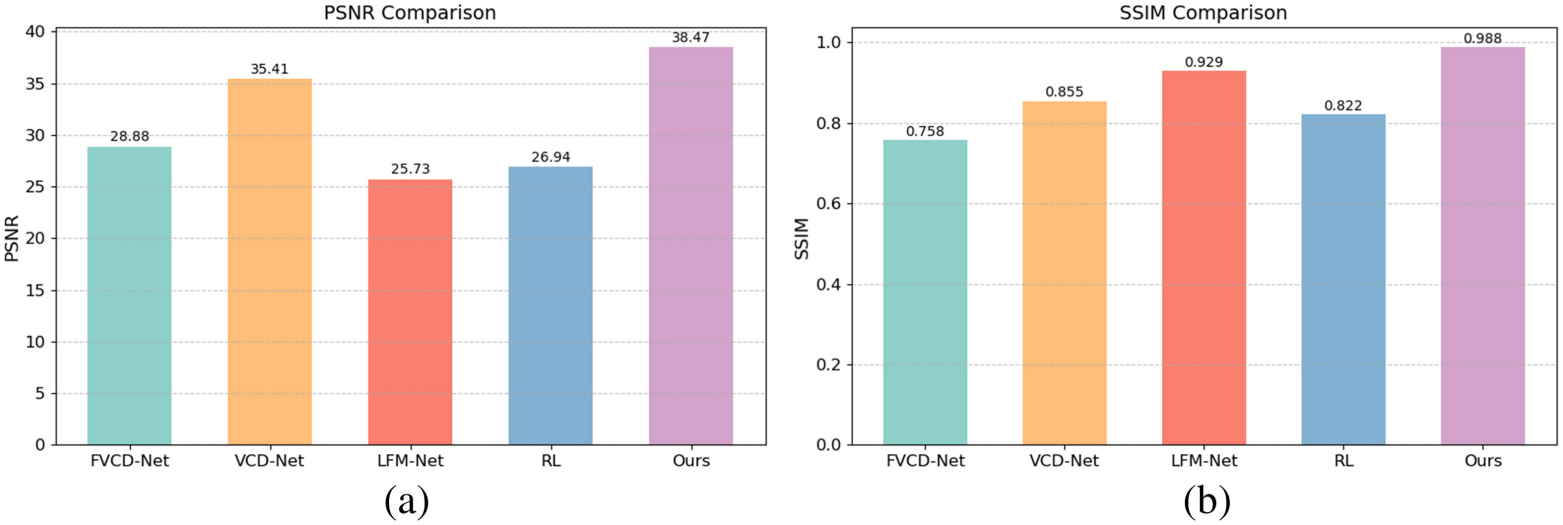
Comparison between different LF-based reconstruction methods: (a) PSNR and (b) SSIM.

To validate the proposed algorithm, we compared it with several existing LF-based 3D reconstruction algorithms, including deep-learning-based methods, such as the FLFM-based FVCD-Net^38^ and the LFM-based VCD-Net^19^ and LFM-Net,^17^ as well as the traditional iterative optimization algorithm RL.^9^ Figure 5 presents the results from the quantitative analysis of these algorithms, highlighting that the proposed method outperformed the existing algorithms in all metrics. Specifically, the traditional RL algorithm exhibited an evident disadvantage compared with the deep learning methods, which indicates that it struggles to handle complex 3D reconstruction tasks. Although FVCD-Net and VCD-Net performed relatively well on single-modality LF data, their PSNR and SSIM values remained suboptimal due to their insufficient utilization of information. LFM-Net achieved a better SSIM, which indicates that it possesses advantages in structural fidelity; however, its relatively low PSNR score indicates limited overall reconstruction quality. By comparison, the proposed method excelled in terms of both the accuracy and consistency of reconstruction, achieving the highest PSNR and SSIM. This underscores the effectiveness of the multimodality fusion approach in leveraging complementary information to achieve superior 3D reconstruction.

### 3.4 Depth Estimation Analysis

**Fig. 6:**
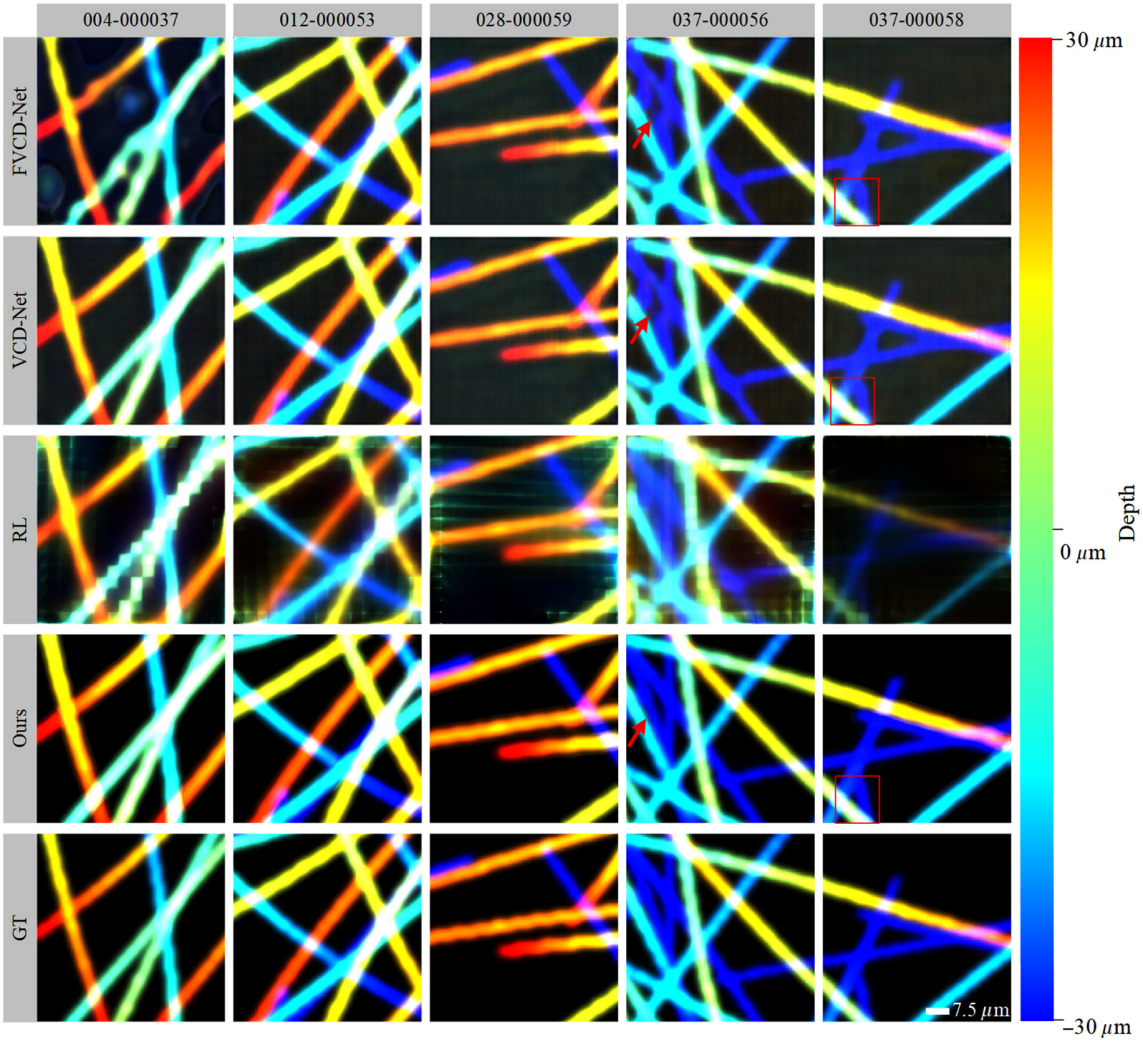
Comparison of depth estimation maps between LF-based reconstruction methods.

Figure 6 demonstrates the depth estimation results for the compared algorithms. The visualized depth estimation maps reveal significant biases in depth prediction for the traditional RL algorithm and the single-modal methods, i.e., FVCD-Net and VCD-Net. Specifically, the RL method and FVCD-Net exhibited noticeable deviations in edge-detail recovery and depth distribution. Although VCD-Net performed relatively well, its depth predictions showed inconsistent brightness levels and occasional structural discontinuities. The inconsistent color distribution suggests that these methods struggle to map depth information accurately, which leads to deviations in depth perception. In addition, the structural discontinuities indicate an inability to maintain spatial coherence and continuity in complex structures. These issues highlight the limitations of single-modal methods in simultaneously achieving accurate depth estimation and spatial consistency. However, the proposed multimodal approach provided depth estimates highly consistent with the GT in terms of color distribution, structure restoration, and edge details, which underlines its superiority in volume reconstruction. The precise color mapping and structural continuity underscore the effectiveness of our method in addressing the limitations of single-modal approaches.

### 3.5 Frequency Analysis

**Fig. 7:**
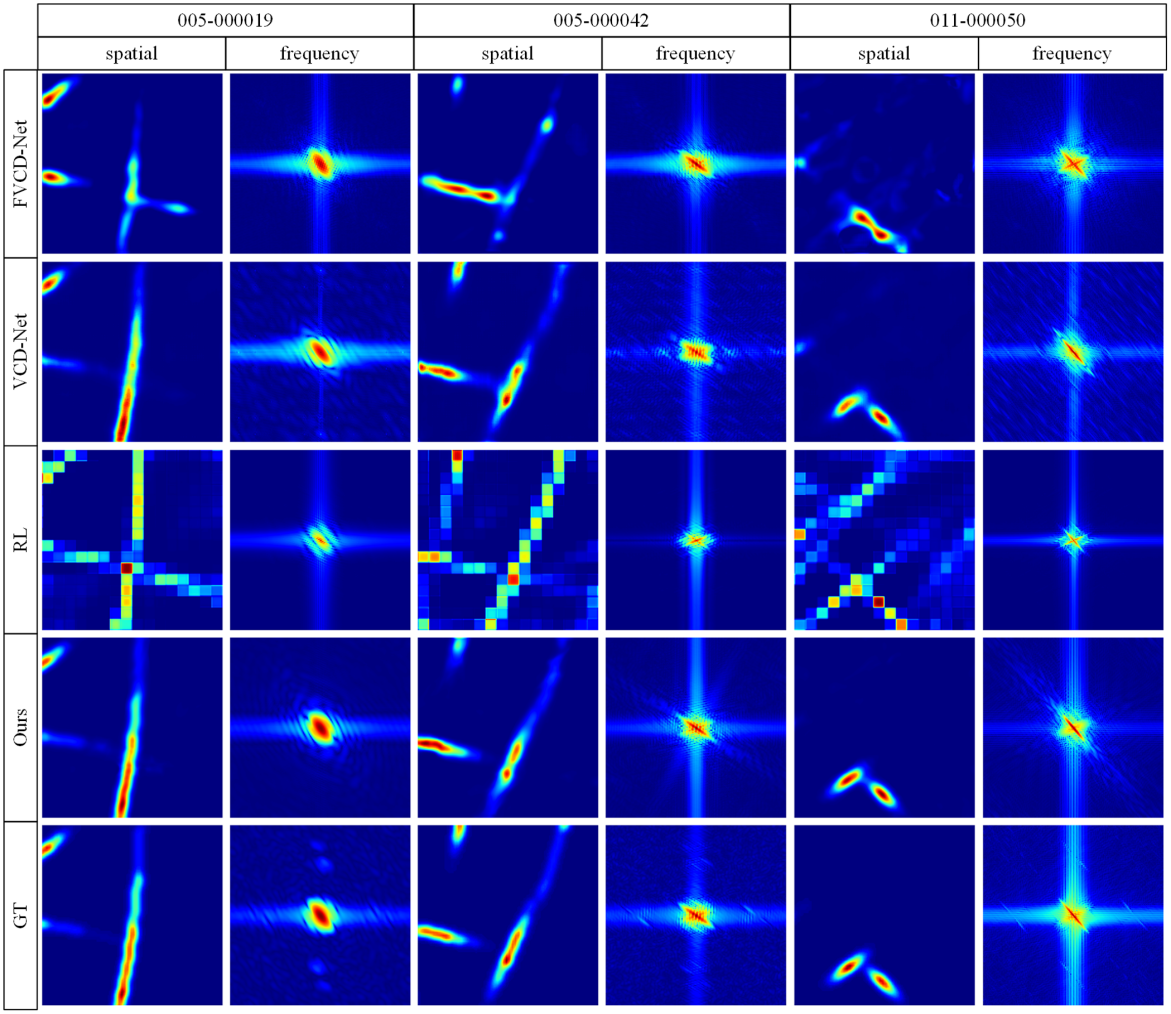
Comparison of frequency images between LF-based reconstruction methods.

To identify further differences between the proposed method and the other methods, we compared them in terms of the spatial frequency analysis of slices (Fig. 7). Based on the observed spatial domain slices, the proposed method demonstrated superior reconstruction quality, with clearer structures and better-preserved fine details, particularly in terms of sharp edges and the continuity of elongated features. In the frequency domain, our approach displayed the most balanced frequency response, preserving strong low-frequency energy (indicative of global structural integrity) and well-distributed high-frequency components, especially along diagonal and axial directions, which reflect edge sharpness and texture orientation. The spectrum was highly symmetric and continuous, with clear directional patterns, resembling those of the GT, which indicates effective preservation of anisotropic structures. By contrast, RL suffered from spectral artifacts and aliasing, which manifested as irregular blocky patterns in the frequency maps. In addition, the maps of VCD-Net and FVD-Net exhibited weakened high-frequency content and lacked directional components, resulting in blurred reconstructions with compromised texture fidelity. Thus, the superior spectral characteristics of the proposed method are essential for enhancing image clarity and structural accuracy. These findings highlight the advantage of our method in reconstructing images that are not only visually sharp but also structurally faithful in both the spatial and frequency domains.

### 3.6 Ablation Study

To verify the effectiveness of each branch in the proposed multimodal LF reconstruction network, we conducted an ablation study that involved testing the input from each modality and observing its impact on the final reconstruction quality. Table 1 presents the average performance metrics for each combination of branches on the test dataset compared with the GT. When using only LFM, FLFM, EPIs, or a fusion of different modalities as input, the PSNR and SSIM values indicated moderate performance. This suggests that single-modality input (LFM, FLFM, or EPIs alone) provides limited information, hindering the reconstruction of high-precision images that simultaneously capture spatial and depth information. However, when dual-modality images were used as input, the reconstruction performance improved significantly. The proposed multimodality approach greatly enhanced the restoration of image details, compensating for the limitations of single-modality inputs in terms of either angular or spatial resolution. The proposed method achieved a PSNR of 38.4729 and an SSIM of 0.9876, offering significant improvement over the other combinations of modalities. In terms of computational efficiency, reconstruction of the selected dataset (176×176×61  pixels) requires only ∼0.0472  s on a single NVIDIA RTX 4090 GPU. Thus, the proposed method effectively leverages the complementary strengths of each modality, delivering reconstruction outcomes that closely approximate the GT in terms of both detail and overall structure.

**Table 1:** Comparison of performance with different combinations of modalities as input.

Method	PSNR	SSIM
LFM	28.1378	0.9668
FLFM	29.9357	0.9490
LF_EPI	29.2013	0.9246
FLF_EPI	28.2681	0.9028
LFM+FLFM	34.4376	0.9715
LFM+LFM_EPI	34.4294	0.9781
FLFM+FLFM_EPI	35.5629	0.9526
LFM+LFM_EPI+FLFM	36.7556	0.9842
Ours (LFM+LFM_EPI+FLFM+FLFM_EPI)	38.4729	0.9876

**Fig. 8:**
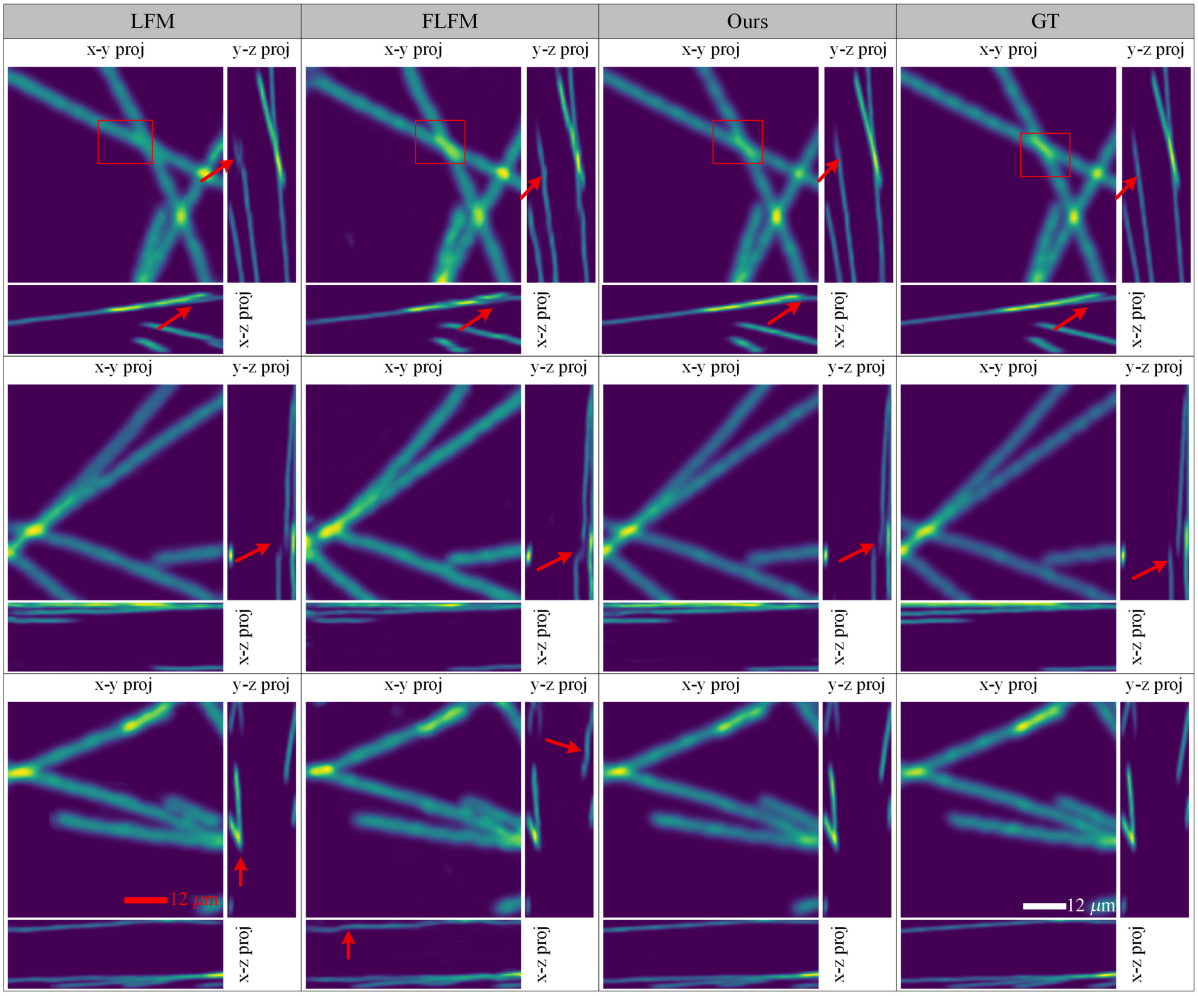
Visualization of projection maps with different modalities.

To verify the superiority of the proposed algorithm in depth reconstruction, we evaluated the projection maps of the reconstructed results. Figure 8 presents the projection heatmaps on the X−Y, X−Z, and Y−Z planes obtained using LFM, FLFM, and the proposed method. Regarding the X−Y projections, the proposed method achieved the closest match to the GT in terms of both consistency of intensity and the smoothness of structural contours. The reconstructions obtained using only LFM input appeared smoother but suffered from inconsistent intensity distributions and poor edge clarity (e.g., row 1). By contrast, using only FLFM input yielded a more uniform intensity distribution across the structures but introduced noticeable roughness and discontinuities in the texture. This suggests that FLFM contributes more to spatial detail recovery, whereas LFM provides smoother global structures. However, the proposed method combines these strengths effectively. In the X−Z and Y−Z projections, depth-related artifacts became more prominent. Using only FLFM input resulted in distorted or fragmented depth features (e.g., bent lines in row 1, Y−Z), which indicate insufficient depth information. Although using LFM-only input did not result in such bending, structural inconsistencies were observed (e.g., row 1, Y−Z). These results suggest that LFM input is more effective in preserving angular information, which is critical for depth recovery. By comparison, the intensity distributions of the proposed multimodality method in the projection maps closely matched those of the GT. Moreover, the straight-line features in the GT were preserved in our reconstruction, which confirms the accuracy of the proposed method in reconstructing both spatial and depth information.

**Fig. 9:**
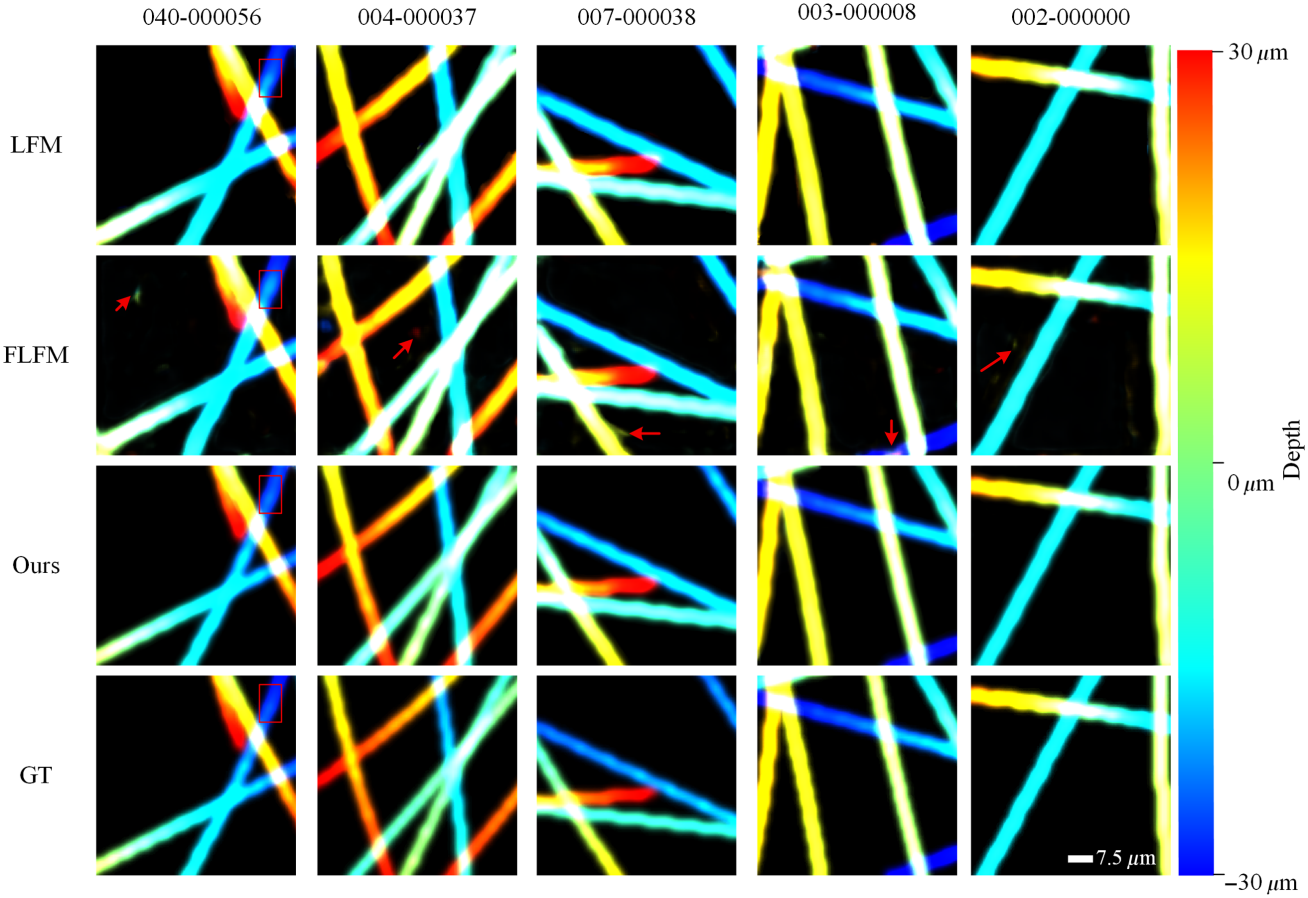
Visualization of depth estimation maps with different modalities.

Furthermore, to evaluate the effectiveness of depth estimation, the reconstructed volumes were visualized by representing different depths with different colors, as shown in Fig. 9. Using only LFM input resulted in overly thickened synthetic tubulin structures, likely due to depth ambiguity or defocus-induced blurring, which led to pseudo-structural spreading. By contrast, FLFM-only reconstructions showed scattered false points that did not exist in the GT, which indicates inaccurate depth localization caused by limited angular information. Notably, in regions where the tubulins were spatially close or overlapping, the single-modality reconstructions exhibited inconsistent color distributions, not at the overlap itself, but in its immediate surroundings. This suggests that the limited angular or spatial cues in single-modality inputs fail to resolve depth ambiguities in complex regions, causing feature spreading. Overlapping structures likely confuse the depth estimation component of the network, which results in erroneous diffusion of features across adjacent layers. These artifacts manifest as false color transitions or blurred contours near the overlap zones, underscoring the limitations of single-modality approaches in complex 3D configurations. By contrast, the proposed multimodality method produced reconstructions that closely matched the GT in both color and structural detail. These visualization results verify the effectiveness of multimodality fusion in achieving precise and reliable 3D reconstructions.

### 3.7 Validation on Mouse Brain Blood Vessels Dataset

**Fig. 10:**
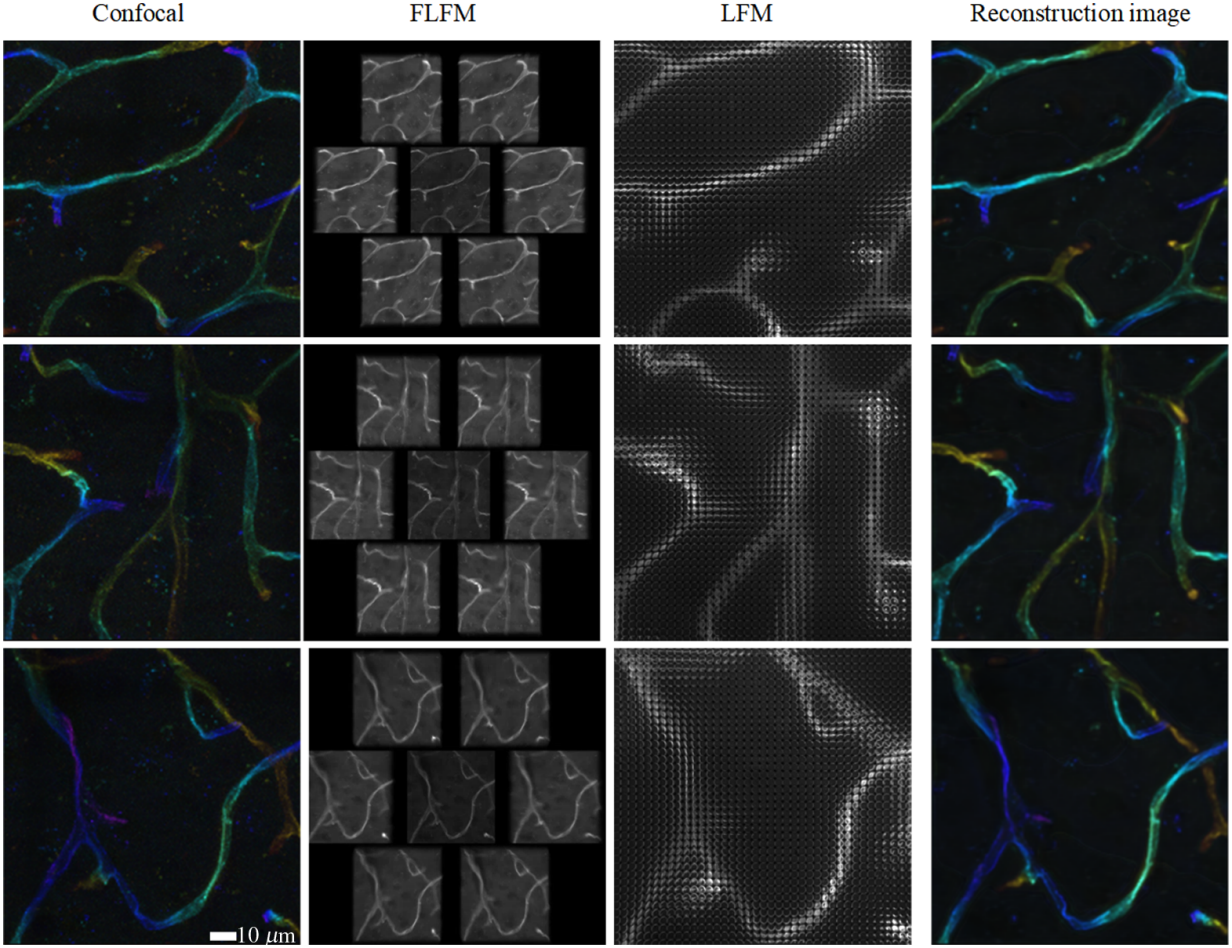
Visualization of mouse brain vessels.

To further validate the method on complex biological structures, we also studied the mouse brain blood vessels dataset.^17^ This dataset was acquired via confocal microscopy with the following specifications: individual volume dimensions of 1287×1287×64  pixels, 362 samples in total, and a volume size of 112×112×57.6  μm. The imaging was performed using a 40×/1.3 NA oil immersion objective, and stained with tomato lectin (DyLight594 conjugated, DL-1177, Vector Laboratories, Newark, California, United States). The ground truth images show intricate vascular networks with varying diameters, complex branches, and three-dimensional connectivity that pose significant challenges for reconstruction algorithms. From these high-quality confocal images, we simulated a corresponding FLFM image. In Fig. 10, the 3D confocal ground truth, the LFM images (also from the reported open source data^17^), simulated FLFM images, and reconstructed 3D volumes based on our proposed method are displayed. From the visualization comparison, it can be seen that the intricate vascular structures have been effectively reconstructed. Notably, the reconstruction successfully captures and preserves the morphology and continuity of larger vessels, with their primary branching patterns clearly delineated. Quantitative evaluation reveals PSNR a value of 35.0548 and structural SSIM a value of 0.8424, respectively. These results confirm that our method maintains high performance across diverse datasets beyond synthetic microtubules.

## 4 Conclusion

To address the limitations of single-modality methods in terms of resolution, depth information recovery, and reconstruction quality, this paper proposed a 3D imaging method that combines the advantages of FLFM and LFM. By leveraging the complementary properties of multimodal images, the proposed 3D reconstruction model fuses the high angular resolution of LFM images, the high spatial resolution of FLFM images, and the parallax information from both their EPIs. Simulation results based on two open source datasets confirm that this dual-modality approach delivers enhanced structural detail, more accurate depth estimation, and better spatial continuity compared with single-modality baselines. Quantitatively, for the synthetic tubulins dataset, it achieved a PSNR of 38.4729 and an SSIM of 0.9876, markedly surpassing single-modality methods. The improvements were especially evident in complex regions, such as overlapping or closely spaced synthetic tubulins, where single-modality inputs tended to introduce artifacts or miss subtle details. The experimental results highlight the effectiveness of leveraging complementary angular and spatial information, verifying the suitability of the proposed method for dynamic and high-throughput 3D imaging applications. The reconstruction results for the mouse brain blood vessels dataset further demonstrate that the proposed method can address the unique challenges posed by real biological samples with their inherent structural complexity.

To go beyond the simulation study based on the open source datasets, we are working on building a dual-branch optical system with both functions of LFM and FLFM (as described in Fig. S1 in the Supplementary Material). We are then able to apply the proposed reconstruction method to the real dual-modal experiment data. Once the fusion neural network is trained on the experimental optical parameters along with the experiment data, the method demonstrated here is expected to be still effective. Our method offers the potential to be extended to more complex scenarios, such as real-time 3D imaging of 100 to 300  μm biological specimens (e.g., subcellular organelles, cellular dynamics, small model organisms) with appropriate optical parameters and will provide a more efficient and reliable solution for applying the light field imaging principle across diverse fields.

## Supplementary Material

10.1117/1.JBO.31.3.036002.s01

## Data Availability

The code used in this study will be published on https://github.com/yyy-ou/LFM-FLFM-Net to ensure reproducibility. Data and materials are available upon request from the authors.
