# Supplementary material for: Improved 3D image reconstruction via deep-learning-based fusion of light-field microscopy and Fourier light-field microscopy images
Source: J Biomed Opt. 2026 Mar 3;31(3):036002. doi: 10.1117/1.JBO.31.3.036002 (PMC12955040; doi:10.1117/1.JBO.31.3.036002)
Supplement: Supplementary file 1 [file JBO_031_036002_SD001.pdf]

Supplementary information

I. Experiment Setup Under Construction

The results presented in the main text were obtained by simulations based on the parameters adpated from the recently reported light-field microscopy studies. The major purpose is to conduct our research on a solid ground to verify the theoretical feasibility and robustness of our proposed fusion type reconstruction method. However, we are also processing to build a dual-branch imaging setup that integrates light field microscopy (LFM) and Fourier light field microscopy (FLFM) such that we can realize experimentally the application of our novel reconstruction method. As shown in Fig. S1, the hybrid optical system is based on a common optical objective with the magnification 50x and the NA 0.7, and is built upon an Olympus IX73 inverted microscope platform. The light source is an LED with the 525 nm central wavelength (Thorlabs SOLIS-525C) .

(1)FLFM Branch

As shown on the top of Fig. S1, the FLFM branch utilizes the DOIT3DMicro Fourier light field imaging module, which integrates a tube lens (f_t=180 mm), a Fourier lens (f_{FL}=100 mm), and a microlens array (f_{MLA}=6.4 mm, 1000 μm pitch), and a CMOS sensor (with the pixel size 2.2 μm). With this branch, we actually have performed 3D imaging of butterfly wing structures, and will publish the results elsewhere. The FLFM branch provides a calculated Field of View (FOV) of about 312.5 μm and a Depth of Field (DOF) of about 12.6 μm.

(2)LFM Branch

As shown on the right side of Fig. S1, it is the LFM branch which is still under upgrading and constructing. The light path is directed through a tube lens (f_t=180 mm) towards a microlens array (MLA). While a 300 μm pitch MLA was initially used, we are replacing it by one with higher pitch (Thorlabs MLA150-7AR-M, with a 150 μm pitch, a 10 mm effective array size, and a 5.2 mm effective focal length) in order to achieve higher spatial resolution. The image will be captured by a color CMOS camera (LIT.Neon10) with a sensor size of 7.4 mm × 5.55 mm and a resolution of 4000×3000 pixels (1.85 μm pixel size). Due to the limited size of the CMOS sensor relative to the size of the MLA, we will employ a relay lens system (Thorlabs MAP1030100-A, f_1=100 mm, f_2=30 mm) to achieve a 3× image shrinking. This design will yield a calcualted FOV of about 200 μm, and a alculated DOF of about 310 μm.

This dual-branch optical design is intended to support subsequent experimental work, and to directly apply our proposed fusion type reconstruction. This optical system will have some different optical parameters comparing to our current simulation study, and our proposed fusion type neural network will be trained on these parameters and the new experimental data. It is expected that this hybrid optical system along with the fusion type reconstruction will achieve high quality 3D imaging for diverse biological samples.

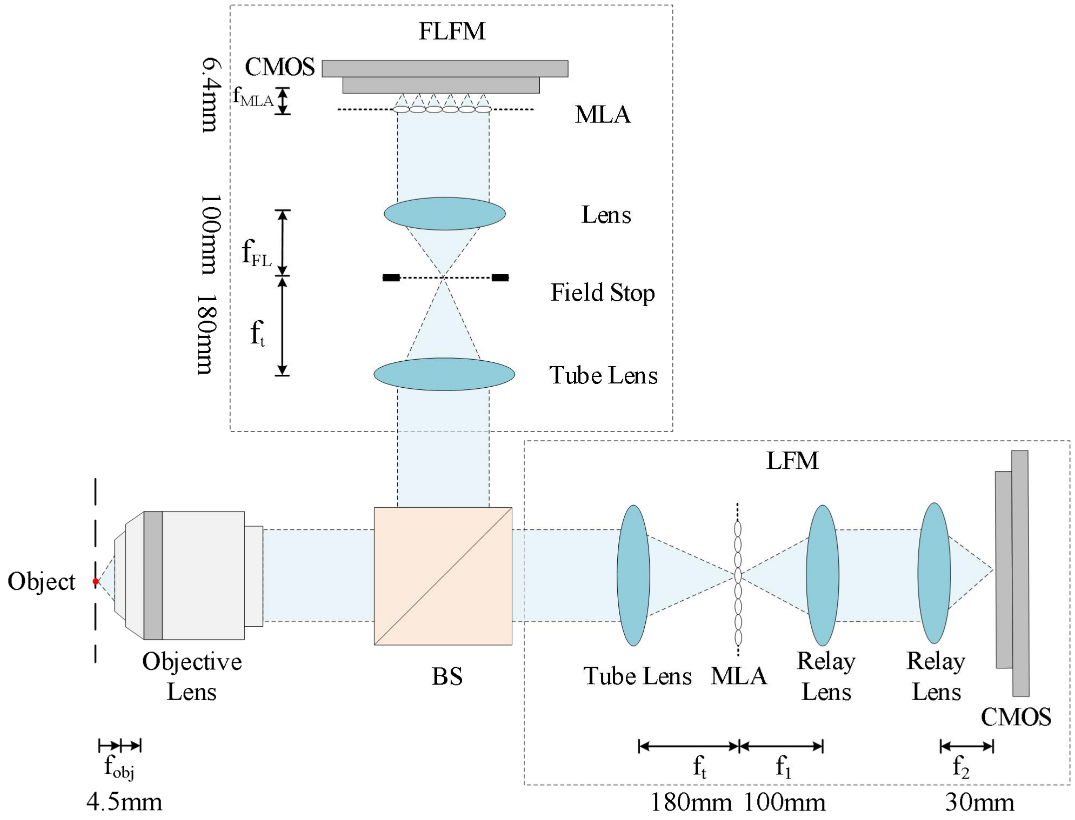

Fig. S1 Schematic of the dual-branch LFM–FLFM experiment setup under construction
